# Myasthenia Gravis With Reversible Pyramidal Tract Damage and Pseudo Internuclear Ophthalmoplegia. A Case Report and Literature Review

**DOI:** 10.3389/fneur.2019.00957

**Published:** 2019-09-10

**Authors:** Yinghong Weng, Yan Min, Zhenghe Sheng, Jia Li, Dehong Huang

**Affiliations:** ^1^Department of Neurology, Guangzhou Hospital of Traditional Chinese Medicine Affiliated With Guangzhou University of Chinese Medicine, Guangzhou, China; ^2^Department of Traditional Chinese Medicine, Liuzhou People's Hospital, Liuzhou, China; ^3^Department of Neurology, China-Japan Union Hospital of Jilin University, Changchun, China

**Keywords:** myasthenia gravis, pseudo internuclear ophthalmoplegia, pyramidal tract damage, signs and symptoms, prognosis

## Abstract

Myasthenia gravis (MG) is a rare and treatable antibody-mediated autoimmune disease. Pseudo internuclear ophthalmoplegia (-INO) or pyramidal tract damage is rarely observed in MG, and there were no known cases of MG with both pseudo-INO and pyramidal tract damage. Here, we report a case of a 61-year-old female suffering from MG accompanied by pseudo-INO and pyramidal tract damage with a rapid progressive course. Her blood and cerebrospinal fluid (CSF) tests were normal, except for the presence of the anti-acetylcholine receptor antibody. CT and contrast enhancement of the chest showed a thymic involution. MRI and contrast enhancement images of the brain and whole spine were normal. Both the clinical response to the administration of neostigmine and the repetitive nerve stimulation test were positive. The motor evoked potentials at lower limb recordings were normal. According to her signs, symptoms, decrementing response on repetitive stimulation test, elevated anti-acetylcholine receptor antibody and positive response to neostigmine, the patient was diagnosed as having MG. After treatment with pyridostigmine, intravenous immunoglobulin, prednisone acetate tablets and methotrexate, all her symptoms disappeared, including pseudo-INO and pyramidal tract damage. To our best knowledge, this is the first report of a case of MG with both pseudo-INO and pyramidal tract damage. Based on our case and a review of the literature, we propose that pyramidal tract damage and pseudo-INO can be two signs of MG, and that MG can cause damage to other systems besides neuromuscular junctions.

## Introduction

Myasthenia gravis is a rare autoimmune disease caused by specific antibodies mostly targeting the anti-acetylcholine receptor antibody (AChR-Ab), leading to fluctuating fatigability and skeletal muscle weakness (1, 2). It can appear at any age and affect more than 700,000 people around the world (3). Patients with MG suffer from extreme fatigue and can develop considerable disability. However, it is a treatable disease. If the correct diagnosis is made early and standardized treatments are available, MG can be controlled very well in many patients (4). But due to the many rare manifestations of MG, it is difficult for clinicians to recognize, leading to delays in diagnosis and treatment. However, any delay can affect the treatment effect and even the residual disability. Therefore, we decided to report this rare case of MG, in order for clinicians to better grasp its clinical manifestations.

Internuclear ophthalmoplegia (INO) is a disorder of conjugate horizontal gaze. It is caused by damage to the medial longitudinal fasciculus (MLF) (5). INO-like eye movements without MLF lesions have been called pseudo-INO (6). Pseudo-INO rarely occurs in MG. Moreover, pyramidal tract damage is also rarely reported in MG. Here, we report a case of MG accompanied by both pseudo-INO and pyramidal tract damage with a rapid progressive course. To our best knowledge, this is the first report of MG with both pseudo-INO and pyramidal tract damage.

## Case Presentation

A 61-year-old Asian female was admitted to our department due to 5 days of blepharoptosis and diplopia. The symptoms spread rapidly to slurred speech, muscle weakness with difficulty swallowing and fatigue in the extremities, especially after exertion. On examination, she presented with bilateral ptosis, horizontal nystagmus of the right eye, and fixed left eyeball (Video S1), normal vision and fundus, weakness of facial muscles, reduced proximal muscle strength of extremities (3–4 degrees), normal muscular tension, generalized hyperreflexia, ankle clonus, and presence of bilateral Babinski signs. Eyelid and limb fatigue tests were positive. The score of the Quantitative MG scoring system (QMG) was 21, the score of the MG activities of daily living profile (MG-ADL) was 13, and the score of the MG Composite (MGC) was 23.

The examinations for rheumatism, autoimmune-related antibody spectrum, and tumor markers were normal. Routine blood tests, serum immunity markers, ANA, RF, TSH, and anti-thyroid antibody were normal. Radioimmunoprecipitation revealed an AChR-Ab concentration of 3.2 nmol/L with no detectable MuSK-Ab (normal range < 0.04 nmol/L). Anti-ganglioside antibodies were negative. The cerebrospinal fluid (CSF) pressure was 115 mm H_2_O (normal range 80–180 mm H_2_O). CSF routine, biochemical, TORCH10, autoimmune encephalitis test, CSF IgM, CSF IgA, CSF IgG, CSF oligoclonal band, CSF specific IgG oligoclonal band, and 24 h CSF IgG intrathecal synthesis rate were normal. Blood and CSF paraneoplastic markers were normal. There were no bacteria, Cryptococcus or acid-resistant bacilli in the CSF smear.

**Figure 1:**
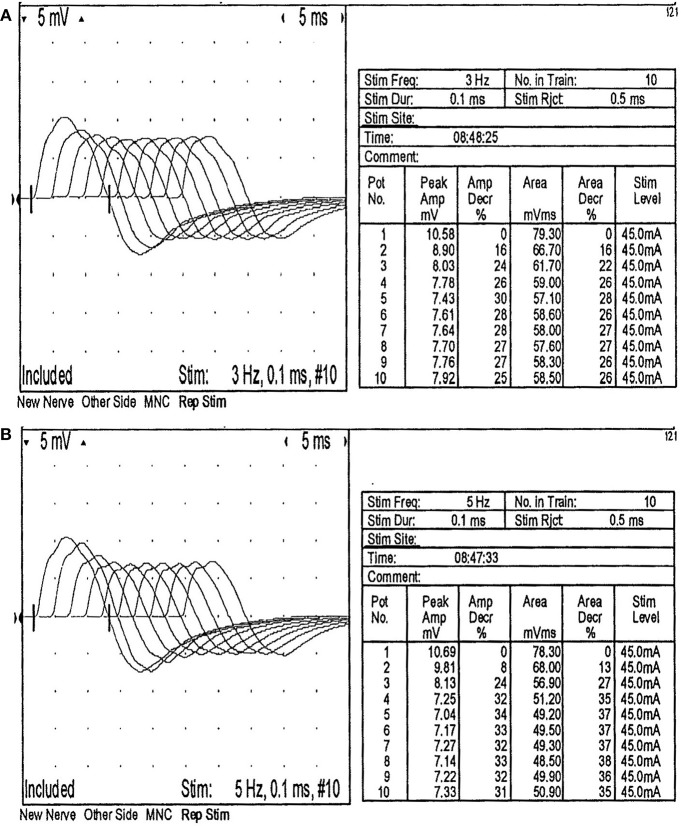
The activity of the right facial nerve in the repetitive nerve stimulation test was decreased. **(A)** The amplitude of the fifth wave was 30% lower than that of the first wave when the right facial nerve was stimulated at 3 Hz repetition frequency. **(B)** The amplitude of the fifth wave was 34% lower than that of the first wave when the right facial nerve was stimulated at 5 Hz repetition frequency.

The neostigmine test was positive. The repetitive nerve stimulation test showed that the amplitude of the low-frequency stimulation of right facial nerve progressively decreased (3 Hz 30%, 5 Hz 34%) (Figure 1). The amplitude of the low-frequency stimulation of right accessory nerve was also decreased progressively (3 Hz 28%, 5 Hz 29%). The nerve conduction tests were normal. The motor evoked potentials at lower limbs recording were normal. CT and contrast enhancement of the chest showed a thymic involution. But the patient didn't undergo thymectomy and had no histopathological results. MRI and contrast enhancement images of the brain and whole spine were normal.

Our final diagnosis was generalized MG (Osserman grade IIB, MGFA grade IIIA). The treatment options were weighed in the context of this patient. She was treated with pyridostigmine and Intravenous immunoglobulin (IVIg). The IVIg dosage was 0.4 g/kg body weight/5 days. After the third day following the course of IVIg, her Babinski signs disappeared, and her limbs were stronger than before. She was subsequently administered prednisone acetate tablets and the dose was increased slowly. After 2 months of treatment, her QMG score was 11, the MG-ADL score was 7, and the MGC score was 9. She was discharged from the hospital on pyridostigmine, prednisone acetate tablets and methotrexate. All the symptoms and positive signs disappeared after 6 months, and there was no disease recurrence after 2 months of follow–up.

This study was approved by the human research ethics committee of the Guangzhou Hospital of Traditional Chinese Medicine affiliated with Guangzhou University of Chinese Medicine. Written informed consent was obtained from the patient who participated in this study.

## Discussion

The patient from this case report showed the typical manifestations of MG, such as fluctuating fatigability and weakness of ocular, bulbar, and limb skeletal muscles. The neostigmine test and AChR-Ab were positive. The repetitive nerve stimulation test showed a decline in the amplitude of the low-frequency stimulation of nerves of more than 15%. CT and contrast enhancement of the chest showed a thymic involution. After treatment with pyridostigmine, IVIg, prednisone acetate tablets and methotrexate, the symptoms improved rapidly, and then disappeared completely. There was no recurrence on follow-up, and the prognosis was good. The diagnosis of MG in this patient was clear, but the patient had nystagmus and weakness of facial muscles, generalized hyperreflexia, ankle clonus, and presence of bilateral Babinski signs which were easy to misdiagnose. To date, many case reports of MG with pyramidal tract damage were diagnosed as MG with multiple sclerosis (MS) (7–9) or MG with neuromyelitis optica spectrum disorder (NMOSD) (10–14). However, our patient's brain MR and CSF OB were normal, which did not support the diagnosis of MS or NMOSD.

Studies in recent years have shown that MG may not only damage the neuromuscular junctions of skeletal muscles, but may also involve the CNS (15–17), peripheral nervous system (18) and autonomic nervous system (19, 20).

Vaknin-Dembinsky et al. (11) studied 24 patients with MG for evident signs of CNS involvement, and found that the incidence of CNS involvement in MG was higher than previously reported. Sharma et al. (21) recorded one case of MG with reversible pyramidal tract signs and thyrotoxic antibodies. The patient had a protrusion of the eyes, ptosis, diplopia, weakness in the masticatory muscles, problems with swallowing, extremities and bladder, hyperreflexia and absent abdominals, and bilateral extensor plantar responses. The neostigmine test and electromyography together with thymic hyperplasia confirmed the diagnosis of MG. After 4 days of treatment with neostigmine and carbimazole, the patient's upper motor neuron signs became normal. Pang et al. (22) described a 40-year-old patient with acute onset MG involving pyramidal tract damage. The patient had diplopia, weakness in the muscles for swallowing, face and extremities, hyperreflexia of lower extremities, and presence of the left Babinski sign. Positive AChR-Ab and RNS confirmed the diagnosis of MG. After he was treated with IVIg and pyridostigmine for 16 days, his Babinski sign disappeared. In summary, both our patient and the other reported cases showed weakness of the extraocular, bulbar, facial, cervical and limb muscles, which was most severe in the evening, as well as pyramidal tract damage. There was no evidence of other CNS lesions. The positive signs disappeared after treatment with cholinesterase inhibitors, prednisone acetate tablets, IVIg, and immunosuppressive agents. Most of the reports were subacute or chronic. Only one report had acute onset, but no nystagmus. However, our patient's disease was rapidly progressive, with acute onset and nystagmus.

The first case of pseudo-INO in MG was reported by Glaser in 1966 (23). Nijsse et al. (24) reported a case of ocular MG with pseudo-INO in one eye, without CNS damage. The AChR-Ab test was positive, and the symptoms resolved after treatment with pyridostigmin. Acers et al. (25) described a case of ocular MG with horizontal nystagmus. The course of the disease was longer than 1 month. The neostigmine test was positive. The symptoms disappeared completely after neostigmine treatment. Ito et al. (6) reported a case of MG complicated by bilateral pseudo-INO. The patient had fluctuating diplopia, tinnitus and imbalance, followed by fluctuating dysphagia, and fatigue of both upper limbs. The AChR-Ab test was positive. Cholinesterase inhibitors relieved the symptoms and the eye movements became normal after thymectomy. Khanna et al. (26) studied 2 cases of ocular MG with horizontal nystagmus. In MS patients, the peak velocity of MG level nystagmus was similar to or slightly faster than that of healthy people, and the nystagmus velocity was the slowest when MS was adducted. In conclusion, the patients described in the discussed reports and our patient had no other CNS lesions, and the symptoms disappeared after treatment with cholinesterase inhibitors or thymectomy. Most of the reports described ocular MG with a chronic course. However, our patient had generalized MG with horizontal nystagmus, which progressed rapidly to the bulbar muscle. The severity of the disease required the clinicians to quickly identify and correctly diagnose the disease and formulate a precise individualized treatment program.

The mechanism of MG with reversible pyramidal tract damage is still unclear, with two main theories. There are mainly two viewpoints. One viewpoint claims that MG is a kind of receptor disease, which may not only be limited to neuromuscular junctions, but also involve other systems. In 1979, it was verified that the AChR-Ab was intrathecal (27) based on the specific values observed in the CSF of patients with MG and IgG and AChR-Ab in the serum. Mavra et al. (28) found the positive oligoclonal IgG in the CSF in patients with MG. They thought that MG may be associated with immune abnormalities within the CNS, and further investigations with more sophisticated techniques may provide insights into the immune events within the CNS underlying the pathophysiology of MG. It may be the combination of AChR-Ab and nicotinic AChR (n-AChR) in the central nervous system that impedes the integration of Ach and n-AChR to generate a series of immune reactions (29). The other view declares that the underlying cause may be a generalized autoimmune response. Zhang et al. (30) discovered that the concentration and synthesis rate of intrathecal IgG in the CSF of patients with CNS involvement was higher than in MG patients without pyramidal tract signs or healthy people. It was speculated that the pyramidal tract signs are caused by the immune response related to the pyramidal tract pathway due to the synthesized intrathecal IgG. Vaknin-Dembinsky et al. (11) discovered the presence of anti-AQP4 antibodies in MG with evident signs of CNS involvement. They speculated that an autoimmune response targeting AQP4 may be an integral part of the immunopathogenetic mechanism of MG.

The underlying mechanisms causing nystagmus in MG are not fully understood. Khanna et al. (26) considered that the reason might be intrassaccadic neuromuscular fatigue or selective sparing of pale global fibers. Nijsse et al. (6, 24) also thought it might help to overcome the adduction weakness of the opposite eye, which was consistent with Hering's law of equal innervation.

## Conclusions

To our best knowledge, there are no reports of MG accompanied by both pyramidal tract damage and nystagmus. We think that reversible pyramidal tract damage and pseudo-INO were two of the rare signs of MG. With the continuous exploration of MG, we consider that MG may be an AchR-Ab-based autoimmune disease that can involve the entire nervous system, and not just a single type of neuromuscular junction, also involve the CNS (15–17), peripheral nervous system (18) and autonomic nervous system (19, 20). Recently, the Osserman and MGFA classification schemes (31), which are commonly used in the clinical assessment of MG, do not include clinical manifestations other than the involvement of neuromuscular junction. With the accumulation of data from clinical observations of MG, we hope that the diagnostic and therapeutic guidelines for MG will broaden the classification to other nervous systems. Therefore, clinicians can better grasp the clinical manifestations of MG, avoid misdiagnosis and missed diagnosis, and better guide the treatment.

## Data Availability

All datasets generated for this study are included in the manuscript/Supplementary Files.

## Ethics Statement

All procedures were approved by the ethics committee of Guangzhou Hospital of Traditional Chinese Medicine affiliated with Guangzhou University of Chinese Medicine. Our patient provided written informed consent.

## Author Contributions

YW contributed intellectual content by drafting the manuscript, including medical writing. YM, ZS, and JL contributed by revising the manuscript, including medical writing. DH contributed by revising the manuscript, including medical writing, and diagnosing the patient. All authors read and approved the final manuscript.

### Conflict of Interest Statement

The authors declare that the research was conducted in the absence of any commercial or financial relationships that could be construed as a potential conflict of interest.

## Supplementary Material

Video S1 Nystagmus of the right eye and fixed left eye, representing a pseudo-INO in a patient with generalized MG. Note the slight ptosis of both eyes.
